# Clinical usefulness of repeated sputum culture for the identification of pneumonia pathogens: A retrospective study

**DOI:** 10.1371/journal.pone.0351167

**Published:** 2026-06-10

**Authors:** Ji Hyun Yun, Jiho Park, Hyun Kyun Ki

**Affiliations:** Division of Infectious Diseases, Department of Internal Medicine, School of Medicine, Konkuk University, Konkuk University Medical Center, Seoul, Republic of Korea; Gulu University, UGANDA

## Abstract

Sputum culture is essential for identifying pneumonia pathogens, but its sensitivity remains limited. This study evaluated the clinical utility of repeated sputum cultures and factors associated with detecting additional significant isolates in subsequent cultures. We retrospectively analyzed adult patients with pneumonia who underwent three serial sputum cultures at a tertiary hospital between January 2021 and December 2022. We excluded patients with over 48 h interval between each sputum sample or those with coronavirus disease 2019. Initial and subsequent culture results were compared, and clinical factors associated with detecting additional significant isolates were evaluated. A total of 132 patients were included. Sputum quality (p = 0.71) and culture positivity rates (p = 0.25) were similar across serial cultures. Fungi, particularly *Candida* species, were more frequently isolated from subsequent cultures (p = 0.02). The cumulative detection rate of significant isolates did not improve, although the cumulative number was higher in third than initial cultures (p = 0.01). Hypertension, inappropriate empirical antibiotics, shorter intervals to repeat culture, and greater pneumonia severity were independent risk factors for detecting additional significant isolates. Serial sputum cultures provided limited additional diagnostic information and were not associated with meaningful improvement in the sputum quality, culture positivity, or cumulative detection of significant isolates.

## Introduction

Sputum culture is an important laboratory test for identifying the pathogens responsible for pneumonia. In Korea, pneumonia is one of the leading causes of death, and its burden is particularly high among older adults and patients with underlying comorbidities [1]. The American Thoracic Society and Infectious Diseases Society of America guidelines for community-acquired pneumonia (CAP) recommend sputum culture for patients at risk of resistant pathogens or those requiring hospitalization [2]. Similarly, Korean guidelines recommend sputum culture and urinary antigen testing for *Streptococcus pneumoniae* and *Legionella* species in hospitalized patients with CAP, and blood culture in severe pneumonia [3]. In Korea, *Staphylococcus aureus*, *S. pneumoniae*, and gram-negative bacilli, including *Pseudomonas aeruginosa* and *Klebsiella pneumoniae*, have been reported as major causative pathogens of CAP [3,4]. In a meta-analysis, the sensitivity of all sputum cultures was 36% (22–53%), regardless of sputum quality, and 73% (26–96%) when only good-quality samples were analyzed [5]. Wide variations in sputum culture sensitivity are mainly attributed to prior antibiotic use, differences in sample quality, and delayed specimen processing [6,7]. Therefore, adequate sputum quality and timely sampling are essential for improving culture sensitivity. Nevertheless, the overall diagnostic yield of sputum culture remains suboptimal.

To overcome these limitations, non-culture-based tests, such as urinary antigen tests and polymerase chain reaction (PCR), have been introduced [8,9]. These methods can enhance the pathogen detection, guide antibiotic selection, and reduce inappropriate antibiotic use [10]. However, they have limitations, including the inability to provide antibiotic susceptibility results and higher costs compared with culture-based methods. Therefore, sputum culture remains an important diagnostic tool in clinical practice.

In pulmonary tuberculosis, repeated sputum examination is an established strategy to improve diagnostic sensitivity, and current guidelines recommend obtaining multiple sputum specimens [11–13]. However, unlike tuberculosis, bacterial pneumonia is usually treated with immediate antimicrobial therapy after specimen collection, which may rapidly reduce culture yield. Although repeated sputum cultures are sometimes performed in routine clinical practice, their additional diagnostic value in bacterial pneumonia remains uncertain. Clarifying this issue may help identify low-value testing and support diagnostic stewardship in clinical practice.

In this study, we evaluated repeated sputum culture results to determine whether it improves pathogen detection in pneumonia. Moreover, we investigated clinical factors associated with the identification of significant isolates on subsequent sputum cultures.

## Materials and methods

### Study design

We enrolled adult patients with pneumonia who were admitted to a tertiary hospital in Seoul, Republic of Korea, between January 2021 and December 2022. Patients with International Classification of Diseases (ICD)-10 codes relevant to pneumonia and available sputum culture results were screened. Their medical charts were retrospectively reviewed. We included patients who underwent three consecutive sputum cultures (at admission and twice subsequently). The decision to perform repeated sputum cultures was made at the discretion of the attending physician as part of routine clinical care, rather than according to a hospital or study protocol. Only hospitalized adult patients were included in this study. Patients were excluded if the initial sputum culture was not obtained within 48 h of admission; if subsequent cultures were performed more than 48 h apart; if there was no clinical evidence of pneumonia within 48 h of admission; or if patients were diagnosed with coronavirus disease 2019 (COVID-19).

The results of the initial, second, and third sputum cultures were analyzed. The sputum sample quality, culture positivity rates, and identified isolates were compared across serial cultures. The cumulative rates and counts of significant isolates were also compared. Additionally, subgroup analyses restricted to good-quality sputum samples and expectorated sputum samples were performed to assess the potential influence of sputum quality and specimen collection method on the results. Clinical factors associated with identifying additional significant isolates in subsequent sputum cultures, which were not detected in previous cultures, were analyzed.

### Definitions

Pneumonia was diagnosed when radiologic evidence of pulmonary infiltration (X-ray or computed tomography) was accompanied by at least two of the following clinical criteria: i) body temperature of ≥38°C or <36°C; ii) white blood cell count of ≥11,000 or <4,000/μL; and iii) presence of purulent sputum or endotracheal aspiration. Sputum samples were graded using the modified Murray–Washington grouping system [14]. Samples classified as Group 4 (epithelial cells 10–25/low-power field [LPF] and white cells > 25/LPF) or Group 5 (epithelial cells <10/LPF and white cells >25/LPF) were considered good quality. Sputum quality was considered improved if an initially poor-quality sample met these criteria in subsequent cultures. Microorganisms were categorized as Gram-positive cocci (GPC), Gram-positive bacilli (GPB), Gram-negative bacilli (GNB), or fungi. Isolates, such as viridans streptococci, *Enterococcus* species, *Candida* species, and *Corynebacterium striatum*, were considered contaminants. Microorganisms not classified as contaminants were considered significant isolates. COVID-19 testing was performed using quantitative real-time reverse transcription PCR (Real-Q 2019-nCoV Detection Kit, BioSewoom, Seoul, Republic of Korea). Respiratory virus testing—including adenovirus, bocavirus, seasonal coronavirus, influenza, human metapneumovirus, parainfluenza virus, respiratory syncytial virus, and rhinovirus—was performed using PCR (Real-Q RV Ⅱ Detection Kit, BioSewoom).

Clinical factors—including age, sex, underlying comorbidities, pneumonia severity, and empirical antibiotic use—were reviewed. Pneumonia was classified as community-acquired or hospital-acquired. Chronic lung diseases included bronchiectasis, tuberculosis sequelae, asthma, and chronic obstructive pulmonary disease. Ischemic heart disease, heart failure, arrhythmias, and valvular heart disease were categorized as chronic heart diseases. Liver cirrhosis and hepatitis were classified as chronic liver diseases. Patients with chronic kidney disease or end-stage renal disease were categorized as having chronic renal disease. Patients who had received steroids, immunosuppressants, chemotherapy, or radiation therapy within 1 month before admission were considered immunosuppressed. Pneumonia severity was assessed using the CURB-65 score, with severe pneumonia defined as a CURB-65 score ≥3. Patients who were intubated within 7 days of admission were classified as having undergone intubation. The appropriateness of empirical antibiotic therapy was evaluated based on initial sputum culture results and was classified as appropriate if identified pathogens were susceptible, inappropriate if they were non-susceptible, and not assessable if no microorganisms were identified or if isolates were considered contaminants.

### Sputum culture

Sputum samples were obtained by expectoration or tracheal aspiration and immediately transferred to the laboratory. Samples were stored at 4–8℃ and inoculated onto blood agar and MacConkey agar plates regardless of sputum quality. Incubation was performed at 37℃ with 5% CO₂ for up to 24 h. Identification and antibiotic susceptibility testing were conducted using the VITEK^®^2 system (bioMérieux, Hazelwood, MO, USA).

### Statistical analysis

The chi-square and Fisher’s exact tests were used for categorical variables to evaluate differences between groups. The t-test and Mann–Whitney U test were used to compare continuous variables. Differences were considered statistically significant when the p-value was < 0.05. Statistical significance for serial sputum culture comparisons (e.g., initial vs. second sputum culture) was set at p < 0.017 using the Bonferroni method. Trends in the cumulative number of significant isolates were assessed using a generalized linear mixed model. Logistic regression analysis was performed to identify factors associated with detecting additional significant isolates in subsequent sputum cultures. Statistical analyses were performed using SPSS software (version 29.0; SPSS Inc., Armonk, NY, USA) and R software (version 4.3.2, R Foundation for Statistical Computing, Vienna, Austria).

### Ethical approval and consent to participate

The hospital’s Institutional Review Board (IRB No. 2023-04-077) approved this study. The informed consent requirement was waived owing to the study’s retrospective nature. Clinical data were accessed for research purposes between 31/05/2023 and 31/12/2024. During data collection, the authors had access to identifiable patient information, which was anonymized prior to analysis.

## Results

### Patients

**Fig 1:**
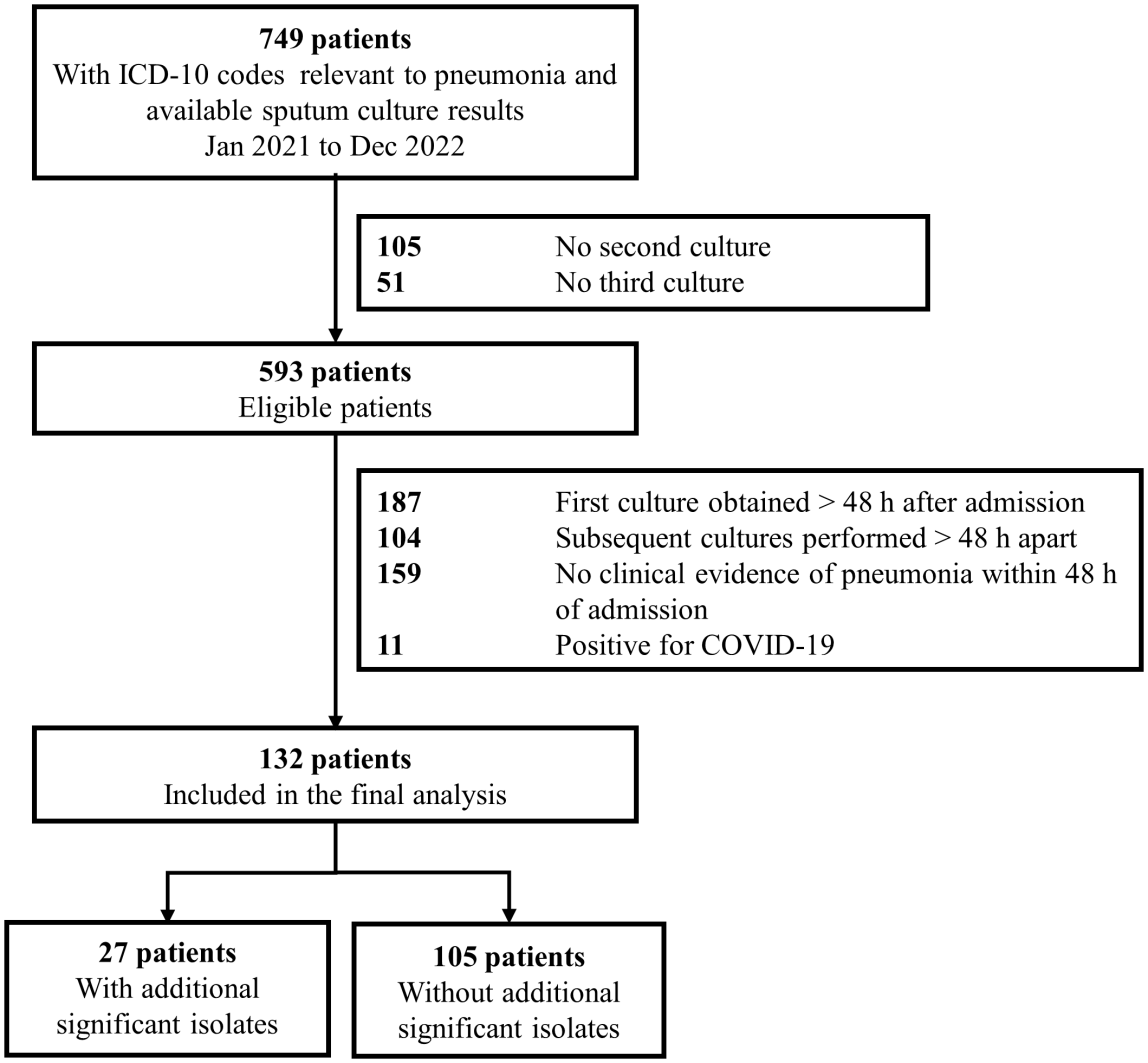
Flowchart of the study population. During the study period, 749 patients with ICD-10 codes relevant to pneumonia and available sputum culture results were screened. Of these, 105 and 51 patients, respectively, from whom a second and third sputum culture was not obtained, were excluded. The remaining 593 patients were eligible for the study. After excluding 187 patients whose first sputum culture was obtained > 48 h after admission, 104 patients whose subsequent sputum cultures were performed > 48 h apart, 159 patients without clinical evidence of pneumonia within 48 h of admission, and 11 patients who tested positive for COVID-19, 132 patients were included in the final analysis. Among them, 27 patients had additional significant isolates identified in subsequent sputum cultures, whereas 105 did not. **Abbreviations:** ICD, International Classification of Diseases; COVID-19, coronavirus disease 2019.

During the study period, 749 patients with ICD-10 codes related to pneumonia and available sputum culture results were screened. Among them, 187 patients underwent initial sputum culture more than 48 h after admission, 105 did not undergo second sputum culture, 51 did not undergo third sputum culture, 104 underwent subsequent sputum culture more than 48 h apart, 159 lacked clinical evidence of pneumonia within 48 h of admission, and 11 were tested positive for COVID-19. The remaining 132 patients were included in the analysis (Fig 1).

### Comparison between serial sputum cultures

There were 46 (35%) good-quality sputum samples in the initial cultures, 47 (36%) in the second, and 41 (31%) in the third, with no significant differences among groups (p = 0.71). Sputum quality improved in 27 patients (21%) in the second culture and in 19 patients (14%) in the third culture. The median interval from admission to culture was 1 day (interquartile range [IQR], 1–1) for the initial culture, 2 days (IQR, 1–2) for the second culture, and 3 days (IQR, 2–3) for the third culture. Positive culture results were observed in 48 (36%) initial cultures, 60 (46%) second cultures, and 59 (45%) third cultures, with no significant differences between groups (p = 0.25). *S. aureus* and *P. aeruginosa* were the most common GPC and GNB isolates, respectively. Most fungi were identified as *Candida* species, except for four *Aspergillus* isolates. Detection rates of GPC and GNB were similar across serial cultures (p = 0.77 for GPC; p = 0.99 for GNB). However, fungi were detected more frequently in subsequent cultures (p = 0.02), particularly in the third culture compared with the initial culture (p = 0.005). No significant differences were observed in either the rate or cumulative rate of significant isolates detection across serial sputum cultures (p = 0.85 and p = 0.09, respectively) (Table 1). The median interval from the initiation of empirical antibiotic therapy to the second and third sputum cultures was 2 and 3 days, respectively. Antibiotic therapy had been changed from the initial empirical regimen before the second and third sputum cultures in 17 (13%) and 39 (30%) patients, respectively. Microbiological outcomes did not differ significantly according to whether empirical antibiotic therapy had been changed before subsequent sputum cultures (S1 Table). Nonetheless, the cumulative number of significant isolates increased by approximately 1.6-fold in the third sputum culture compared with the initial culture (p = 0.01), whereas this trend was not observed for the second culture (Table 2).

**Table 1:** Comparison between initial, second, and third sputum culture results.

	Initial n = 132	Second n = 132	Third n = 132	p-value
Good-quality sputum	46 (35)	47 (36)	41 (31)	0.71
Quality improvement		27 (21)	19 (14)	
Interval from admission to culture, days, median (IQR)	1 (1–1)	2 (1–2)	3 (2–3)	
Interval from the initiation of empirical antibiotic therapy to culture, days, median (IQR)	1 (0–1)	2 (1–2)	3 (2–3)	
Tracheal aspirate	24 (18)	32 (24)	37 (28)	0.16
Positive culture results	48 (36)	60 (46)	59 (45)	0.25
Gram-positive cocci	10 (8)	11 (8)	8 (6)	0.77
*Staphylococcus aureus*	6	9	7	
*Streptococcus pneumoniae*	2	0	1	
*Streptococcus agalactiae*	1	1	0	
*Enterococcus faecium*	1	1	0	
Gram-negative bacilli	28 (21)	27 (21)	27 (21)	0.99
*Pseudomonas aeruginosa*	14	13	11	
*Klebsiella pneumoniae*	2	3	7	
*Acinetobacter* spp.^a^	3	4	3	
*Stenotrophomonas maltophilia*	1	2	3	
*Enterobacter cloacae*	2	1	1	
*Escherichia coli*	3	1	2	
*Proteus* spp.^b^	3	3	2	
*Serratia marcescens*	1	1	0	
*Klebsiella aerogenes*	0	1	1	
Gram-positive bacilli	0	1 (1)	1 (1)	0.61
*Corynebacterium striatum*	0	1	1	
Fungi	13 (10)	25 (19)	30 (23)	0.02
*Candida albicans*	10	17	24	
*Candida* spp. other than *C. albicans*	2	7	7	
*Aspergillus* spp.	1	2	1	
Polymicrobial^c^	4 (3)	7 (5)	10 (8)	0.26
Significant isolates	37 (28)	38 (29)	34 (26)	0.85
Cumulative significant isolates	37 (28)	46 (35)	54 (41)	0.09

Data are presented as no. (%), unless otherwise indicated

Abbreviation: IQR, interquartile range

^a^*A. baumannii* was identified in nine cases and the species was not identified in one case.

^b^*P. mirabilis* was identified in six cases and *P. vulgaris* in two cases.

^c^Polymicrobial infections were identified in four initial cultures, seven second cultures, and 10 third cultures. The organisms detected in polymicrobial infections were as follows:

Initial culture (four cases): *S. marcescens* and *P. aeruginosa*; *S. aureus* and *P. aeruginosa*; *S. aureus* and *Candida* spp. (non-*albicans*); *E. faecium* and *C. albicans* (each one case)Second culture (seven cases): *S. aureus* and *C. albicans* (two cases); *S. marcescens* and *P. aeruginosa* (one case); *S. aureus* and *P. aeruginosa* (one case); *K. aerogenes* and *C. albicans* (one case); *P. aeruginosa* and *S. maltophilia* (one case); *C. albicans* and *Candida* spp. (non-*albicans*) (one case)Third culture (10 cases): *S. aureus* and *P. aeruginosa* (two cases); *S. aureus*, *C. albicans*, and *Candida* spp. (non-*albicans*) (one case); *K. aerogenes* and *C. albicans* (one case); *P. aeruginosa* and *S. maltophilia* (one case); *E. coli* and *Candida* spp. (non-*albicans*) (one case); *K. pneumoniae*, *P. aeruginosa*, and *S. maltophilia* (one case); *C. albicans* and *Candida* spp. (non-*albicans*) (one case); *S. maltophilia* and *Candida* spp. (non-*albicans*) (one case); *S. pneumoniae* and *C. albicans* (one case)

**Table 2:** Cumulative number of significant isolates in serial sputum cultures using a generalized linear mixed model.

	Estimate	p-value
Initial sputum culture	Reference	
Second sputum culture	0.268	0.21
Third sputum culture	0.526	0.01

When only good-quality sputum samples were analyzed, positive culture rates between the initial and subsequent cultures remained similar (17 [37%] vs. 23 [49%] vs. 22 [54%], p = 0.12). *S. aureus* and *P. aeruginosa* were the most common GPC and GNB isolates. Significant isolates were identified in 14 (30%), 18 (38%), and 13 (32%) of the initial, second, and third cultures, respectively, and did not significantly differ (p = 0.88) between serial cultures (S2 Table). An additional subgroup analysis restricted to expectorated sputum samples was performed to assess the potential influence of specimen collection method (S3 Table). In this analysis, no significant differences were observed in sputum quality, culture positivity, or detection and cumulative detection of significant isolates across serial sputum cultures. However, fungi were more frequently identified in subsequent sputum cultures (p = 0.01), particularly in the third sputum culture (p = 0.003).

Additional isolates not identified in prior sputum cultures were detected in 28 (21%) of the second and 24 (18%) of the third cultures. *S. aureus* was the most common GPC isolate, while *K. pneumoniae* and *P. aeruginosa* were predominant among the GNB isolates. Most fungal isolates were *Candida* species. Among the additional isolates, significant isolates were identified in 13 (46%) and 14 (58%) of the second and third cultures, respectively (Table 3).

**Table 3:** Characteristics of additional isolates identified in subsequent sputum cultures.

Characteristics	Second, n = 132	Third, n = 132
Additional isolates identified	28 (21)	24 (18)
Gram-positive cocci	6 (5)	3 (2)
*S. aureus*	5	2
*S. pneumoniae*	0	1
*E. faecium*	1	0
Gram-negative bacilli	7 (5)	10 (8)
*P. aeruginosa*	2	2
*K. pneumoniae*	1	6
*Acinetobacter* spp.^a^	2	0
*S. maltophilia*	1	2
*P. mirabilis*	0	1
*K. aerogenes*	1	0
Gram-positive bacilli	1 (1)	1 (1)
*C. striatum*	1	1
Fungi	14 (11)	11 (8)
*C. albicans*	8	8
*Candida* spp. other than *C. albicans*	5	3
*Aspergillus* spp.	1	1

Data are presented as no. (%), unless otherwise indicated.

^a^*A. baumannii* was identified in one case and the species was not identified in one case.

### Comparison between patients with and without additional significant isolates

Significant isolates were newly identified in subsequent sputum cultures in 27 (16%) of 132 patients. Age and sex distributions were comparable between the two groups. Most patients (91%) had CAP, which was similar across groups. Hypertension (15 [56%] vs. 28 [27%], p = 0.004) and a history of cerebrovascular accident (CVA) (8 [30%] vs. 8 [8%], p = 0.005) were more common in the group with additional significant isolates. These patients also had higher CURB-65 scores (p = 0.02) and shorter intervals from admission to the initial culture and from the initial to the second culture (p = 0.03 and p = 0.04, respectively). No significant differences were observed in intubation status, prior antibiotic use, sputum sample quality, or quality improvement between the groups. However, inappropriate empirical antibiotics were administered more frequently (8 [30%] vs. 10 [10%], p = 0.01) in the group with additional significant isolates (Table 4).

**Table 4:** Comparison of characteristics between the patients with and without additional significant isolates in subsequent sputum cultures.

Characteristics	Total, n = 132	Additional significant isolates, n = 27	No additional significant isolates, n = 105	p-value
Age, years, median (IQR)	62 (53–66)	65 (57–67)	61 (53–66)	0.26
Male	98 (74)	22 (82)	76 (72)	0.34
CAP	120 (91)	24 (89)	96 (91)	0.71
Underlying diseases				
Hypertension	43 (33)	15 (56)	28 (27)	0.004
Diabetes mellitus	42 (32)	10 (37)	32 (31)	0.51
Chronic lung diseases	31 (24)	8 (30)	23 (22)	0.40
Chronic heart diseases	18 (14)	5 (19)	13 (12)	0.53
Chronic liver diseases	11 (8)	0	11 (11)	0.12
Chronic renal diseases	14 (11)	4 (15)	10 (10)	0.48
Rheumatologic diseases	7 (5)	2 (7)	5 (5)	0.63
Hematologic malignancy	4 (3)	1 (4)	3 (3)	>0.99
Solid organ cancer	23 (17)	6 (22)	17 (16)	0.57
History of CVA	16 (12)	8 (30)	8 (8)	0.005
Immunosuppressed status	26 (20)	6 (22)	20 (19)	0.71
CURB-65				0.02
0	31 (24)	2 (7)	29 (28)	
1	33 (25)	5 (19)	28 (27)	
2	36 (27)	12 (44)	24 (23)	
3	21 (16)	5 (19)	16 (15)	
4	9 (7)	2 (7)	7 (7)	
5	2 (2)	1 (4)	1 (1)	
Severe pneumonia (CURB-65 ≥ 3)	32 (24)	8 (30)	24 (23)	0.46
Intubation status	39 (23)	9 (33)	30 (29)	0.63
Prior antibiotics usage	37 (28)	7 (26)	30 (29)	0.79
Interval to culture, days, median (IQR)				
Admission to initial	1 (1–1)	1 (1–1)	1 (1–1)	0.03
Initial to second	1 (0–1)	0 (0–1)	1 (0–1)	0.04
Initial to third	2 (1–2)	1 (1–2)	2 (1–2)	0.13
Good-quality sputum				
Initial	46 (35)	11 (41)	35 (33)	0.47
Second	47 (36)	11 (41)	36 (34)	0.53
Third	41 (31)	8 (30)	33 (31)	0.86
Quality improvement	38 (29)	9 (33)	29 (28)	0.56
Respiratory virus infection	5 (4)	0 (0)	5 (5)	0.58
Relevance of empirical antibiotics				
Appropriate	21 (16)	5 (19)	16 (15)	0.77
Inappropriate	18 (14)	8 (30)	10 (10)	0.01

Data are presented as no. (%), unless otherwise indicated

Abbreviations: IQR, interquartile range; CAP, community-acquired pneumonia; CVA, cerebrovascular accident

### Factors associated with additional significant isolates in subsequent sputum cultures

In the univariate analysis, hypertension (odds ratio [OR], 3.44; 95% confidence interval [CI], 1.44–8.24), history of CVA (OR, 5.11; 95% CI, 1.71–15.28), higher CURB-65 scores (OR, 1.46; 95% CI, 1.04–2.03), shorter intervals from admission to the initial culture (OR, 0.40; 95% CI, 0.17–0.94) and from the initial to the second culture (OR, 0.46; 95% CI, 0.22–0.94), and inappropriate empirical antibiotic use (OR, 4.00; 95% CI, 1.40–11.46) were associated with additional significant isolates (Table 5). Multivariate analysis included these variables, as well as age, intubation status, quality improvement in subsequent sputum samples, and prior antibiotic use. Hypertension (adjusted OR [aOR], 3.94; 95% CI, 1.38–11.19; p = 0.01), higher CURB-65 scores (aOR, 1.96; 95% CI, 1.18–3.26; p = 0.01), inappropriate empirical antibiotic use (aOR, 7.42; 95% CI, 1.94–28.44; p = 0.003), and shorter intervals from admission to the initial culture (aOR, 0.10; 95% CI, 0.02–0.45; p = 0.003) and from the initial to the second culture (aOR, 0.17; 95% CI, 0.06–0.52; p = 0.002) were identified as independent factors associated with detecting additional significant isolates in subsequent cultures.

**Table 5:** Factors for identifying additional significant isolates in subsequent sputum cultures.

Risk factors	Univariate analysis result [OR (95% CI)]	Multivariate analysis result^a^	
		Adjusted OR (95% CI)	p-value
Age	1.02 (0.98–1.07)		
Male	1.68 (0.58–4.85)		
Hypertension	3.44 (1.44–8.24)	3.94 (1.38–11.19)	0.01
History of CVA	5.11 (1.71–15.28)		
Intubation status	1.25 (0.51–3.09)	0.27 (0.06–1.07)	0.06
Quality improvement	1.31 (0.53–3.25)		
CURB-65	1.46 (1.04–2.03)	1.96 (1.18–3.26)	0.01
Inappropriate empirical antibiotics	4.00 (1.40–11.46)	7.42 (1.94–28.44)	0.003
Previous antibiotic use	0.88 (0.34–2.28)		
Interval to initial sputum culture	0.40 (0.17–0.94)	0.10 (0.02–0.45)	0.003
Interval initial to second sputum culture	0.46 (0.22–0.94)	0.17 (0.06–0.52)	0.002

Abbreviations: OR, odds ratio; CI, confidence interval; CVA, cerebrovascular accident

^a^ The model fitted the data well in terms of discrimination (C-statistic = 0.84) and calibration (Hosmer–Lemeshow goodness of fit statistic = 3.98, p *=* 0.86).

## Discussion

In this study, we systematically evaluated repeated sputum culture results in patients with pneumonia. Positivity rates and sputum sample quality did not differ significantly between initial and subsequent cultures. Fungi, particularly *Candida* species, were detected more frequently in subsequent sputum cultures, whereas the cumulative detection rate of significant isolates did not increase. Hypertension, shorter intervals to culture, inappropriate empirical antibiotic use, and higher CURB-65 scores were identified as independent risk factors for the detection of additional significant isolates in subsequent cultures.

The overall sputum culture positivity rate was 42%, consistent with previously reported diagnostic yields [5]. GNB were the most frequently identified organisms, with *P. aeruginosa* being the most common isolate in both initial and subsequent cultures. An Asian meta-analysis reported that GNB were identified in 13% of hospitalized patients with CAP, with *K. pneumoniae* being the most common pathogen [15]. In our study, 59 patients (45%) were at risk for *P. aeruginosa* infection due to chronic lung disease, hematologic malignancy, solid organ cancer, or immunocompromised status, which may have influenced the observed sputum culture results.

Repeated sputum cultures did not improve sputum quality, culture positivity, or the cumulative detection rate of significant isolates. Similar findings were observed even when analyses were restricted to good-quality sputum samples. As all patients received antibiotic therapy after the initial sample was obtained, the sensitivity of subsequent sputum cultures may have been reduced [7]. Sputum culture results may provide clinically relevant information in patients with pneumonia, as suggested by a recent study showing an association between sputum culture results and the duration of hospitalization in CAP [16]. However, our findings indicate that routine repetition of sputum cultures offers limited additional diagnostic value. Additionally, reducing unnecessary repeated sputum cultures may contribute to cost savings, reduce laboratory workload, and improve diagnostic stewardship. Previous studies have demonstrated that diagnostic yield can be improved using alternative sampling methods, such as tracheal aspiration, sputum induction, or bronchial lavage [17–21]. Therefore, rather than repeating sputum cultures, efforts should first be made to obtain an adequate initial respiratory specimen. If the initial sputum culture remains non-diagnostic and the patient does not respond to empirical antibiotic therapy, alternative diagnostic approaches, such as tracheal aspiration, bronchoscopy, or other adjunctive microbiologic tests, including molecular tests, may be considered depending on the clinical situation.

The number of fungal isolates significantly increased in subsequent sputum cultures, with most identified as *Candida* species. Broad-spectrum antibiotics—including piperacillin/tazobactam (administered to 86 patients), third-generation cephalosporins (29 patients), cefepime (two patients), and carbapenems (two patients)—were commonly used. Levofloxacin was administered to 16 patients (three as monotherapy and 13 in combination). Broad-spectrum antibiotics disrupt normal airway microbiota and promote fungal overgrowth [22,23]. Thus, antibiotic-induced dysbiosis may explain the increased detection of *Candida* species in subsequent sputum cultures.

Shorter intervals to culture, inappropriate empirical antibiotic use, and higher CURB-65 scores were independently associated with the detection of additional significant isolates in subsequent sputum cultures. Shorter intervals to sputum collection likely reflect a reduced impact of antibiotic exposure on culture results. Previous studies have shown that sputum culture sensitivity decreases over time following antibiotic administration [7]; therefore, cultures obtained soon after treatment initiation may provide additional microbiological information. Appropriate and timely empirical antibiotic therapy is associated with improved survival and shorter hospital stays [24,25]. Conversely, inappropriate empirical antibiotic use may be less effective and is often associated with the detection of new isolates in subsequent sputum cultures. In patients who fail to respond to initial empirical therapy, repeated sputum cultures may assist in identifying causative pathogens. In cases of severe pneumonia, clinicians may pursue more aggressive diagnostic evaluations, including more frequent intubation and invasive sampling procedures. Moreover, mixed infections are common in severe pneumonia [26], which may further increase the likelihood of detecting additional isolates in subsequent sputum cultures.

This study has several limitations. First, patients diagnosed with pneumonia at >48 h after admission were excluded to minimize the effect of prior antibiotic exposure on culture sensitivity, which may have introduced selection bias. Consequently, most patients had CAP, limiting the generalizability of the findings to hospital-acquired pneumonia. Additionally, the study population was relatively older and predominantly male, which may further limit the generalizability of our findings to other patient populations. Second, distinguishing true infection from colonization, particularly for GNB, was difficult in this retrospective study. Although we performed an additional analysis restricted to good-quality sputum samples, misclassification of colonizing organisms as significant isolates cannot be excluded. Third, as sputum samples were collected serially, superinfection with hospital-acquired pathogens during the sampling interval cannot be excluded. Fourth, the relatively small number of patients included in the analysis of factors associated with additional isolates may have limited statistical power. Despite these limitations, this study systematically evaluated consecutive sputum cultures and demonstrated no significant improvement in sputum quality, positivity rate, or cumulative detection of significant isolates, despite a numerical increase in recovered isolates. These findings suggest that the clinical utility of repeated sputum cultures in bacterial pneumonia is limited and that alternative diagnostic approaches may be considered when the initial sputum culture is non-diagnostic and the clinical response is unsatisfactory.

## Conclusion

The quality of sputum samples, the positivity rate of sputum cultures, and the cumulative detection rate of significant isolates were not improved with repeated sputum cultures. *Candida* species were more frequently isolated in subsequent cultures. Therefore, repeated sputum cultures should be interpreted cautiously, as they provide limited additional information concerning pneumonia pathogens and may not be necessary for all patients.

## Supporting information

S1 Table Comparison of the subsequent sputum culture results according to the modification of empirical antibiotic therapy before the second and third sputum cultures. (DOCX)

S2 Table Comparison of serial sputum culture results with good-quality sputum samples. (DOCX)

S3 Table Comparison of serial sputum culture results of expectorated sputum samples. (DOCX)
