# Supplementary material for: Clinical usefulness of repeated sputum culture for the identification of pneumonia pathogens: A retrospective study
Source: PLoS One. 2026 Jun 10;21(6):e0351167. doi: 10.1371/journal.pone.0351167 (PMC13252770; doi:10.1371/journal.pone.0351167)
Supplement: S1 Table — (DOCX) [file pone.0351167.s001.docx]

**S1 Table. Comparison of the subsequent sputum culture results according to the modification of empirical antibiotic therapy before the second and third sputum cultures.**

|  | **Second sputum culture** | | | **Third sputum culture** | | |
| --- | --- | --- | --- | --- | --- | --- |
|  | **Changed, n=17** | **Unchanged, n=115** | **p-value** | **Changed, n=39** | **Unchanged, n=93** | **p-value** |
| Good-quality sputum | 8 (47) | 39 (34) | 0.29 | 12 (31) | 29 (31) | 0.96 |
| Positive culture results | 8 (47) | 52 (45) | 0.89 | 21 (54) | 38 (41) | 0.17 |
| Significant isolates | 6 (35) | 32 (28) | 0.57 | 11 (28) | 23 (25) | 0.68 |
| Cumulative significant isolates | 7 (41) | 39 (34) | 0.56 | 15 (39) | 39 (42) | 0.71 |
