# Supplementary material for: Clinical usefulness of repeated sputum culture for the identification of pneumonia pathogens: A retrospective study
Source: PLoS One. 2026 Jun 10;21(6):e0351167. doi: 10.1371/journal.pone.0351167 (PMC13252770; doi:10.1371/journal.pone.0351167)
Supplement: S2 Table — (DOCX) [file pone.0351167.s002.docx]

**S2 Table. Comparison of serial sputum culture results with good-quality sputum samples.**

|  | **Initial**  **n=46** | **Second**  **n=47** | **Third**  **n=41** | **p-value** |
| --- | --- | --- | --- | --- |
| Positive culture results | 17 (37) | 23 (49) | 22 (54) | 0.12 |
| Gram-positive cocci | 3 (7) | 4 (9) | 1 (2) | 0.44 |
| *Staphylococcus aureus* | 2 | 3 | 1 |  |
| *Streptococcus pneumoniae* | 1 | 0 | 0 |  |
| *Streptococcus agalactiae* | 0 | 1 | 0 |  |
| Gram-negative bacilli | 10 (22) | 12 (26) | 11 (27) | 0.58 |
| *Pseudomonas aeruginosa* | 3 | 4 | 4 |  |
| *Klebsiella pneumoniae* | 2 | 3 | 3 |  |
| *Acinetobacter* spp.^a^ | 1 | 1 | 2 |  |
| *Stenotrophomonas maltophilia* | 0 | 1 | 1 |  |
| *Enterobacter cloacae* | 0 | 1 | 1 |  |
| *Escherichia coli* | 2 | 1 | 1 |  |
| *Klebsiella aerogenes* | 0 | 1 | 0 |  |
| *Proteus* spp.^b^ | 2 | 1 | 0 |  |
| Gram-positive bacilli | 0 | 1 (2) | 1 (2) | 0.34 |
| *Corynebacterium striatum* | 0 | 1 | 1 |  |
| Fungi | 5 (11) | 8 (17) | 9 (22) | 0.16 |
| *Candida albicans* | 2 | 5 | 8 |  |
| *Candida* spp. other than *C. albicans* | 2 | 1 | 0 |  |
| *Aspergillus* spp. | 1 | 2 | 1 |  |
| Polymicrobial | 1 (2) | 3 (6) | 1 (2) | 0.92 |
| Significant isolates | 14 (30) | 18 (38) | 13 (32) | 0.88 |
| Cumulative significant isolates | 14 (30) | 21 (40) | 17 (42) | 0.28 |

Data are presented as no. (%), unless otherwise indicated.

^a^ *A. baumannii* was identified in three cases and the species was not identified in one case.

^b^ *P. mirabilis* and *P. vulgaris* were identified in two and one case, respectively.
