# Supplementary material for: Clinical usefulness of repeated sputum culture for the identification of pneumonia pathogens: A retrospective study
Source: PLoS One. 2026 Jun 10;21(6):e0351167. doi: 10.1371/journal.pone.0351167 (PMC13252770; doi:10.1371/journal.pone.0351167)
Supplement: S3 Table — (DOCX) [file pone.0351167.s003.docx]

**S3 Table. Comparison of serial sputum culture results of expectorated sputum samples.**

|  | **Initial**  **n=108** | **Second**  **n=100** | **Third**  **n=95** | **p-value** |
| --- | --- | --- | --- | --- |
| Good-quality sputum | 33 (31) | 32 (32) | 22 (23) | 0.34 |
| Quality improvement |  | 20 (20) | 12 (13) |  |
| Interval from admission to culture, days, median (IQR) | 1 (1–1) | 2 (1–2) | 3 (2–3) |  |
| Positive culture results | 37 (34) | 43 (43) | 42 (44) | 0.28 |
| Gram-positive cocci | 9 (8) | 8 (8) | 6 (6) | 0.85 |
| *Staphylococcus aureus* | 5 | 6 | 5 |  |
| *Streptococcus pneumoniae* | 2 | 0 | 1 |  |
| *Streptococcus agalactiae* | 1 | 1 | 0 |  |
| *Enterococcus faecium* | 1 | 1 | 0 |  |
| Gram-negative bacilli | 20 (19) | 17 (17) | 18 (19) | 0.93 |
| *Pseudomonas aeruginosa* | 9 | 9 | 7 |  |
| *Klebsiella pneumoniae* | 2 | 1 | 4 |  |
| *Acinetobacter* spp.^a^ | 1 | 1 | 0 |  |
| *Stenotrophomonas maltophilia* | 1 | 1 | 1 |  |
| *Enterobacter cloacae* | 2 | 1 | 1 |  |
| *Escherichia coli* | 3 | 1 | 2 |  |
| *Proteus* spp.^b^ | 2 | 2 | 2 |  |
| *Serratia marcescens* | 1 | 1 | 0 |  |
| *Klebsiella aerogenes* | 0 | 1 | 1 |  |
| Gram-positive bacilli | 0 (0) | 1 (1) | 0 (0) | 0.36 |
| *Corynebacterium striatum* | 0 | 1 |  |  |
| Fungi | 11 (10) | 21 (21) | 25 (26) | 0.01 |
| *Candida albicans* | 9 | 14 | 20 |  |
| *Candida* spp. other than *C. albicans* | 1 | 6 | 6 |  |
| *Aspergillus* spp. | 1 | 2 | 1 |  |
| Polymicrobial^c^ | 4 (4) | 6 (6) | 8 (8) | 0.37 |
| Significant isolates | 28 (26) | 25 (25) | 23 (24) | 0.96 |
| Cumulative significant isolates | 28 (26) | 32 (32) | 38 (40) | 0.10 |

Data are presented as no. (%), unless otherwise indicated.

^a^ *A. baumannii* was identified in one case and the species was not identified in one case.

^b^ *P. mirabilis* was identified in four cases and *P. vulgaris* in two cases.

^c^ Polymicrobial infections were identified in four initial cultures, six second cultures, and eight third cultures. The organisms detected in polymicrobial infections were as follows:

Initial culture (four cases): *S. marcescens* and *P. aeruginosa*; *S. aureus* and *P. aeruginosa*; *S. aureus* and *Candida* spp. (non-*albicans*); *E. faecium* and *C. albicans* (each one case)
Second culture (six cases): *S. aureus* and *C. albicans* (two cases); *S. marcescens* and *P. aeruginosa* (one case); *S. aureus* and *P. aeruginosa* (one case); *K. aerogenes* and *C. albicans* (one case); *C. albicans* and *Candida* spp. (non-*albicans*) (one case)
Third culture (eight cases): *S. aureus* and *P. aeruginosa* (two cases); *S. aureus*, *C. albicans*, and *Candida* spp. (non-*albicans*) (one case); *K. aerogenes* and *C. albicans* (one case); *E. coli* and *Candida* spp. (non-*albicans*) (one case); *C. albicans* and *Candida* spp. (non-*albicans*) (one case); *S. maltophilia* and *Candida* spp. (non-*albicans*) (one case); *S. pneumoniae* and *C. albicans* (one case)
